# Potential of Inflammatory Protein Signatures for Enhanced Selection of People for Lung Cancer Screening

**DOI:** 10.3390/cancers14092146

**Published:** 2022-04-26

**Authors:** Megha Bhardwaj, Ben Schöttker, Bernd Holleczek, Axel Benner, Petra Schrotz-King, Hermann Brenner

**Affiliations:** 1 Division of Clinical Epidemiology and Aging Research, German Cancer Research Center (DKFZ), 69120 Heidelberg, Germany; b.schoettker@dkfz-heidelberg.de (B.S.); h.brenner@dkfz-heidelberg.de (H.B.); 2 Division of Preventive Oncology, German Cancer Research Center (DKFZ) and National Center for Tumor Diseases (NCT), 69120 Heidelberg, Germany; petra.schrotz-king@nct-heidelberg.de; 3 German Cancer Consortium (DKTK), German Cancer Research Center (DKFZ), 69120 Heidelberg, Germany; 4 Network Aging Research, University of Heidelberg, Bergheimer Strasse 20, 69115 Heidelberg, Germany; 5 Saarland Cancer Registry, Präsident-Baltz-Strasse 5, 66119 Saarbrücken, Germany; b.holleczek@krebsregister.saarland.de; 6 Division of Biostatistics, German Cancer Research Center (DKFZ), 69120 Heidelberg, Germany; benner@dkfz-heidelberg.de

**Keywords:** lung cancer, risk prediction, risk stratification, cancer prevention and screening, smoking exposure, proteomics, LC risk model

## Abstract

**Simple Summary:**

Selection of appropriate high-risk smokers is one of the major challenges for implementing low-dose computed tomography screening for lung cancer. Many lung cancer risk prediction models have been proposed to supplement lung cancer screening. This study evaluated the potential of inflammatory protein markers to enhance lung cancer risk stratification beyond lung cancer risk models. The addition of inflammatory protein markers to existing lung cancer risk models improved risk prediction. The inflammatory protein markers may enhance current risk stratification and may be useful to identify high-risk populations for lung cancer screening.

**Abstract:**

Randomized trials have demonstrated a substantial reduction in lung cancer (LC) mortality by screening heavy smokers with low-dose computed tomography (LDCT). The aim of this study was to assess if and to what extent blood-based inflammatory protein biomarkers might enhance selection of those at highest risk for LC screening. Ever smoking participants were chosen from 9940 participants, aged 50–75 years, who were followed up with respect to LC incidence for 17 years in a prospective population-based cohort study conducted in Saarland, Germany. Using proximity extension assay, 92 inflammation protein biomarkers were measured in baseline plasma samples of ever smoking participants, including 172 incident LC cases and 285 randomly selected participants free of LC. Smoothly clipped absolute deviation (SCAD) penalized regression with 0.632+ bootstrap for correction of overoptimism was applied to derive an inflammation protein biomarker score (INS) and a combined INS-pack-years score in a training set, and algorithms were further evaluated in an independent validation set. Furthermore, the performances of nine LC risk prediction models individually and in combination with inflammatory plasma protein biomarkers for predicting LC incidence were comparatively evaluated. The combined INS-pack-years score predicted LC incidence with area under the curves (AUCs) of 0.811 and 0.782 in the training and the validation sets, respectively. The addition of inflammatory plasma protein biomarkers to established nine LC risk models increased the AUCs up to 0.121 and 0.070 among ever smoking participants from training and validation sets, respectively. Our results suggest that inflammatory protein biomarkers may have potential to improve the selection of people for LC screening and thereby enhance screening efficiency.

## 1. Introduction

With 2.2 million incident cases and 1.8 million deaths in 2020, lung cancer (LC) is the second most common cancer and leading cause of cancer mortality globally [1]. The stage at which LC is diagnosed is crucial, as the 5-year survival is 55% for stage I and less than 5% for stage IV LC cases [2]. Randomized trials have demonstrated that screening heavy smokers by low-dose computed tomography (LDCT) using various definitions of heavy smoking [3,4,5,6,7,8,9,10,11] can reduce LC mortality by up to 30% [5,11]. These LDCT trials preselected heavy smokers as those at highest risk of LC for screening and thereby enhanced the balance of benefits and potential harms [12,13] of screening. Apart from these trial criteria, more refined lung cancer risk prediction models have been developed [14,15,16,17,18,19,20,21] and suggested for enhancing the effectiveness of an LC screening program by enhanced selection of those at highest risk. There is hope that selection of high-risk individuals for lung cancer screening might further be enhanced by biomarkers. Several studies have identified associations between the acute phase inflammatory marker C-reactive protein (CRP) and of cytokines such as interleukin 6 (IL6) and interleukin 8 (IL8) with increased risk of LC [22,23,24,25]. Further enhancement of LC risk prediction might be achieved by combining multiple blood-based protein biomarkers in multi-marker signatures [26,27,28,29]. The aim of this study was to explore if and to what extent inflammatory protein biomarker signatures may enhance selection of those at highest risk for LC. The performance of nine LC risk prediction models individually were compared with combined LC risk model inflammatory protein biomarker signatures.

## 2. Methods

### 2.1. Study Design and Study Population

**Figure 1:**
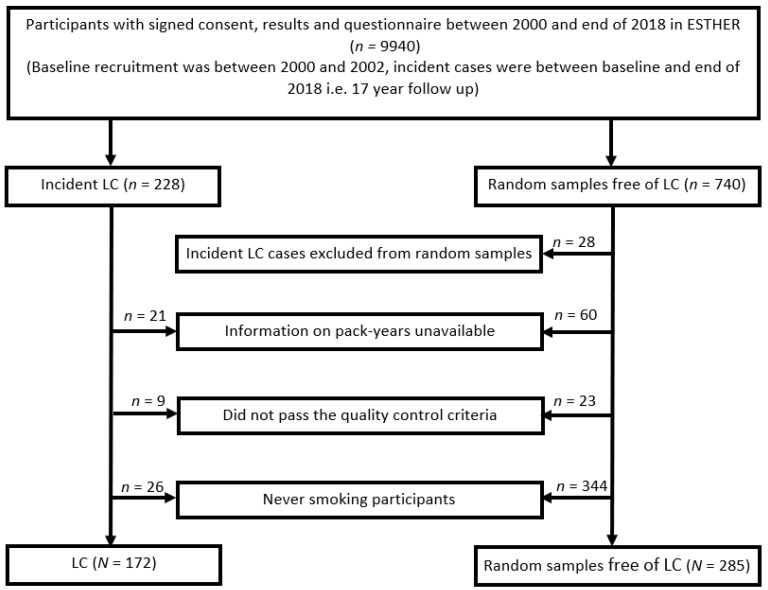
Selection of ever smoking participants from the ESTHER-study. Abbreviations: LC—incident lung cancer; n/N—number.

The protein biomarkers were measured in ever smoking participants from ESTHER, an ongoing population-based cohort study (Full German name: “Epidemiologische Studie zu Chancen der Verhütung, Früherkennung und optimierten Therapie chronischer Erkrankungen in der älteren Bevölkerung”). Details of the ESTHER study have been published elsewhere [30,31]. In brief, participants were recruited between 2000 and 2002 by general practitioners in Saarland, Germany, during a routine health checkup and were followed up with respect to incidence and mortality from major diseases since. At baseline, information was obtained on socio-demographic characteristics, lifestyle factors and health status with standardized self-administered participant and GP questionnaires, and biological samples (blood, stool, and urine) were collected, processed and stored at −80 °C. Prevalence and incidence of cancer at baseline and during follow-up was determined by linking the records with data from Saarland Cancer Registry. By the end of 2018, LC had been diagnosed in 228 participants. In the present study, the protein measurements were performed in all of these 228 incident LC cases and 740 randomly selected LC-free participants. The random samples were selected without any replacement. Derivation and evaluation of algorithms were performed in ever smoking participants exclusively, which comprised 172 LC cases and 285 participants free of LC (Figure 1). The ESTHER study has been approved by the ethics committees of the Medical faculty of Heidelberg University (58/2000) and of the state medical board of Saarland, Germany. Written informed consent was obtained from all participants.

### 2.2. Laboratory Assay

Plasma protein concentrations in the samples were measured utilizing the proximity extension assays (PEA) offered by Olink (Uppsala, Sweden). The full protocol of the PEA has been reported previously [32]. Briefly, the 96 pairs of oligonucleotide-labeled antibodies (92 biomarkers and 4 internal controls) are allowed to bind pairwise to target proteins and when in close proximity a polymerase chain reaction (PCR) reporter sequence is formed due to DNA polymerization which is quantified by real time PCR. For the current study, Olink’s “Inflammation” multiplex panel was used, which allows for simultaneous analysis of 92 biomarkers in 1 µL samples. The full list of protein markers from this panel is provided in Supplementary Table S1. Each assay from this panel has been validated, and information on assay characteristics, such as detection limits, dynamic range, repeatability and reproducibility is available from the manufacturer’s website [33].

### 2.3. Statistical Analysis

The demographic and smoking characteristics of incident LC cases and control participants without LC diagnosis were assessed, and the differences were tested for statistical significance by chi square test. The linear protein values were log transformed to produce normalized protein expression (NPX) and one NPX represents two-fold change in protein concentration. Inflammation biomarkers with >1% of the values below limit of detection (LOD) were excluded from all analyses. The NPX values of each individual protein were compared between LC cases and control participants without LC diagnosis during follow-up using Wilcoxon rank-sum test with adjustment for multiple testing by the Benjamini and Hochberg method [34]. A logistic regression model was used to construct the prediction algorithm for each protein, and the prediction accuracy was evaluated by calculating areas under the ROC curves (AUCs) and their 95% confidence intervals (95% CI).

In order to derive multi-marker algorithms for prediction of incidence of LC, a split-sample approach was used. First, 65% of participants was randomly selected in the training set and the remaining 35% of participants was included in validation set. In the training set, comprising 107 incident LC cases and 190 participants free of LC, smoothly clipped absolute deviation (SCAD) [35] was employed to derive multi-marker algorithms for protein biomarkers only in the form of an inflammation protein biomarker score (INS) and for combined protein biomarkers and self-reported pack-years of smoking (INS-pack-years) score. The performance of these scores for predicting LC incidence was estimated with AUCs not adjusted for overfitting and 95% CI, as well as 0.632+ bootstrap adjusted AUCs (AUC*s) to control for overoptimism [36]. The performance of INS and INS-pack-years models was further evaluated in the validation set consisting of 65 incident LC cases and 95 ever smoking participants free of LC.

Furthermore, we assessed if, and to what extent, the combination of inflammatory protein signatures with LC risk models (“LC risk model-Inf”) could enhance LC prediction by the risk models alone. Nine established LC risk models that are based on slightly different variables as shown in Supplementary Table S2 were included. To derive combined LC risk model-INf algorithms, SCAD and 0.632+ bootstrap was applied on the training set and AUCs for LC risk model only and combined LC risk model-INf algorithms were estimated. The performance of these derived scores were evaluated further in the participants of the validation set. Since algorithms were developed for each LC risk model separately, different algorithms include different numbers and sets of inflammatory biomarkers. DeLong test was performed to assess whether the differences between the AUCs obtained for the LC risk models alone and for the combined LC risk model-INf were statistically significant [37].

To calculate risk prediction of all the nine models, the publicly available R-package (https://dceg.cancer.gov/tools/risk-assessment/lcmodels, accessed on 24 January 2022) was used. All statistical analyses were performed with statistical software R language and environment (version 3.6.3, R core team) [38] using R packages “dplyr”, “glmnet”, “lcmodels”, “ModelGood”, and “pROC”. Statistical testing was two-sided, and *p* values of 0.05 or less were considered to be statistically significant.

## 3. Results

### 3.1. Characteristics of Study Population and Assay Performance

The flow diagram displaying the selection of study participants is provided in Figure 1, and the main characteristics of participants are shown in Table 1. The study included 172 ever smoking LC cases and 285 ever smoking participants that remained free of LC during a mean of 15 years of follow-up. The median age at baseline was 62 and 60 years for LC cases and participants remaining free of LC, respectively. Males represented 71% of LC cases and 62% of the participants free of LC. The proportion of current smokers was much higher among the incident LC cases (62%) as compared to the LC-free participants (35%). The distributions of age, gender and smoking status were similar in the training and the validation sets.

The quality control criteria (QCC) of the biomarker measurements were considered good with 97% of the samples meeting QCC, and the intra-assay and inter-assay coefficient of variances (CV) were 7% and 12%, respectively. When checked for expression, 33 inflammation protein biomarkers that had >1% of the measured values below LOD were excluded from all analyses (marked in grey in Supplementary Table S1).

### 3.2. Predictive Performance of Individual Markers

Mean plasma concentrations of the 59 inflammation proteins in LC cases and participants free of LC are presented in Supplementary Table S3. The differences in mean plasma concentrations between LC cases and participants that remained free of LC were statistically significant (*p* values ≤ 0.05) for 11 proteins; however, after adjustment for multiple testing, only three protein biomarkers displayed statistically significant differences in protein levels (adjusted *p* values ≤ 0.05). Three biomarkers, CUB domain-containing protein 1 (CDCP1), eotaxin (CCL11) and interleukin 12 subunit beta (IL12B), were identified with AUCs ≥ 0.60.

### 3.3. Predictive Performance of Multi-Marker Signatures

To evaluate the performance of multi-marker prediction signatures for comparing LC cases to LC-free controls, SCAD and 0.632+ bootstrap were applied to the 59 inflammation protein biomarkers in participants from the training set. As shown in Table 2, for the prediction of incidence of LC in the training set, an algorithm consisting of nine proteins (“inflammation protein biomarker score”, INS) was identified with AUC* and AUC of 0.770 and 0.771 (95% CI, 0.713–0.828), respectively. The nine inflammatory proteins included in the INS were caspase-8 (CASP8), CCL11, CDCP1, T-cell surface glycoprotein CD8 alpha chain (CD8A), natural killer cell receptor 2B4 (CD244), C-X-C motif chemokine 10 (CXCL10), fibroblast growth factor 19 (FGF19), monocyte chemotactic protein 4 (MCP4) and stem cell factor (SCF).

When SCAD-penalized regression was applied to the 59 protein biomarkers and to the self-reported pack-years of smoking in the training set, an INS-pack-years score was obtained with improved AUC* and AUC of 0.796 and 0.811 (95% CI, 0.760–0.863), respectively. The INS-pack-years score included self-reported pack-years of smoking and the following ten proteins: CASP8, CCL11, C-C motif chemokine 25 (CCL25), CDCP1, CD8A, CD244, CXCL10, C-X-C motif chemokine 9 (CXCL9), FGF19 and matrix metalloproteinase-1 (MMP1). In the validation set, AUCs of 0.742 (95% CI, 0.667–0.818) and 0.782 (95% CI, 0.711–0.854) were observed for INS and INS-pack-years scores, respectively.

The predictive performance of derived scores by LC types in both training and validation sets is reported in Supplementary Table S4. Despite increased random variation, results were rather consistent across types.

### 3.4. Predictive Performance of LC Risk Models and INS, Individually and in Combination

The predictive performances of derived algorithms for each LC risk model alone and in combination with inflammatory protein biomarkers are presented in Table 3. The combined LC risk model-INf algorithms were developed in the training set and were further evaluated in the validation set. In the training set, the Bach model, Pittsburgh predictor, LCRAT and LCDRAT risk models outperformed all other risk prediction models with AUCs of 0.765, 0.767, 0.775 and 0.770, respectively. Improved AUC*s of 0.807, 0.800 and 0.804, respectively, were observed for combined Bach-INf, Pittsburgh predictor-INf and LCRAT-INf algorithms. When performances were evaluated in the validation set, AUCs of 0.770, 0.794 and 0.773 were observed for the three aforementioned combined scores. Overall, the combined LC risk model-INf signatures performed better as compared to LC risk models alone. The addition of inflammatory protein biomarkers to the established nine LC risk models increased AUCs between 0.011 and 0.121 in the training set and between 0.009 and 0.070 in the validation set. The improvement in performance was statistically significant at DeLong *p* value < 0.05 for the combined LLP-INf and PLCOM2012-INf models. Since a separate algorithm was developed for each LC risk model, different sets and numbers of inflammatory protein biomarkers were selected in each combined LC risk model-INf signature. However, several protein biomarkers, such as CDCP1, CD244, CXCL10 and IL8 were included in almost all of the combined algorithms. The INS-pack-years score presented with an AUC of 0.782 in the validation set and the only other LC risk model-INf score performing better was Pittsburg predictor-INf with an AUC of 0.794. For the cases diagnosed within the first 10 years, the Bach-INf outperformed all other models, and at a cutoff yielding 80% specificity, sensitivities of 73% and 60% were observed in the training and validation sets, respectively.

## 4. Discussion

This exploratory study evaluated the predictive performance of blood-based protein markers alone and in combination with pack-years, in a prospective cohort with up to 17 years of follow-up. In the current study, the signature of ten inflammatory protein biomarkers and pack-years of smoking (INS-pack-years) predicted incident lung cancer cases with AUCs of 0.811 and 0.782 in the training and validation sets, respectively. The addition of inflammatory protein biomarkers to the established LC risk models showed improved prediction potential as compared to the LC risk models alone.

The human blood proteome, metabolome, and genome carry great potential for novel approaches to cancer risk prediction and cancer early detection. With emerging technologies for sensitive protein detection even in small sample volumes, standardized multiplex protein detection and quantitation methods are a particularly promising approach in this context. Some of the recent protein detection methods such as PEA are straightforward for routine clinical application as standardized laboratory and statistical data processing procedures have been established. The proximity extension assays used in the current study utilize a pair of oligonucleotide-labeled antibodies or probes that have to be in close proximity for the detection of each protein. PEAs require an exceptionally low sample volume of 1 µL and can detect and quantify protein concentrations with good reproducibility (CV < 15%). The technical assay sensitivity for the PEA assays is in the picogram/ml range, and they can quantify across five logs of abundance. Good assay sensitivity with high target specificity because of dual recognition and requirements of low volume of sample make PEA an efficient method. The inflammation panel used in our study allows for simultaneous detection of 92 circulatory inflammation biomarkers. Inflammation has been associated with both carcinogenesis and tumor progression [39,40]. Chronic inflammation can result from smoking, other exogenous factors, genetic predisposition and occurs in the process of many different diseases. Factors such as severity of disease and adverse pathophysiological changes that can cause acute stress may be associated with elevated levels of several inflammatory markers [41]. Previous studies have provided epidemiological evidence supporting the potential of circulating inflammatory markers for risk prediction of several cancers, especially LC [22,23,24]. The current study identified biomarkers such as CDCP1, CD244 and CXCL10 that were included in INS, INS-packyears and almost all the combined LC risk model-INf signatures. As per the information extracted from the UniProt database [42,43] and presented in the Supplementary Table S5, most of the proteins function as cytokines and are involved in biological processes ranging from angiogenesis to inflammatory response. Lung cancer screening by low-dose computed tomography faces many challenges, and given the differences in health care systems, socio-economic disparities and cultural barriers in different countries, it is essential to develop culture-sensitive screening approaches [44,45,46]. In recent years, besides LDCT trials, many risk models have been proposed for the prediction of lung cancer, and these models include different risk factors such as age, gender, smoking intensity and duration, prior history of lung diseases, occupational exposure to asbestos and family history [14,15,16,17,18,19,20,21]. Seven out of nine risk models such as Bach, Spitz, Hoggart, PLCO_M2012_, Pittsburgh Predictor, LCRAT and LCDRAT, were developed exclusively for ever smokers. The addition of inflammatory protein biomarkers improved the predictive ability of all LC risk models assessed in our study, although the increase in AUCs in the validation set was statistically significant for two of the LC models only, given the small sample size of the validation set. Further validation in larger studies is therefore warranted. Nevertheless, this exploratory study suggests the combination of LC risk models with inflammatory protein scores to be a promising approach for enhanced selection of participants for lung cancer screening programs. The gain in predictive performance would have to be weighed against the additional complexity and costs of risk assessment by the need of blood sampling and analysis. However, such laboratory analyses could be easily embedded in other routine blood sampling commonly employed among older adults in primary care, and blood tests customized to measure these inflammatory proteins could most likely be developed and offered at low cost. Further research should also evaluate the gain in predictive ability and the associated complexity and costs compared to alternative approaches, such as combination of the LC risk models with alternative signatures of proteins [27,29], autoantibodies [47], DNA methylation [48,49], or microRNA [50] biomarkers. Further research should also aim for external validation of the most promising algorithms in independent cohorts.

A major strength of the current study is that the ever smoking participants were selected from a large population-based cohort of older adults who were recruited in the relevant age range for LC screening and followed up with respect to LC incidence over 17 years. However, despite the overall large size of the ESTHER cohort (N = 9940) and the long follow-up, the number of ever smoking participants with incident LC was still rather limited. Utilizing state-of-the-art technology of PEA, 92 circulating inflammatory markers were assessed in 1 µl plasma per sample. Establishment of standardized laboratory procedures for reliable multiplex measurements of proteins even in such small sample volumes should facilitate their implementation in routine medical practice including screening programs. Applying cutting-edge statistical machine learning algorithms, thorough control for overoptimism and internal validation, the markers were evaluated for possible combinations and comparisons with a wide range of established LC risk models. To the best of our knowledge, the INS, INS-pack-years and combined LC risk model-INf scores were evaluated for the first time for long-term prediction of LC in our study. Major limitations include the limited sample size of LC patients, leading to rather wide confidence intervals of the derived indicators of predictive performance. Potential misreporting and recall bias of the smoking variables need careful consideration, although previous biomarker-based validation suggests high accuracy of self-reported smoking in the ESTHER cohort [51]. As the observed improvement of predictive ability with the addition of inflammatory proteins was statistically significant for two of the LC risk models only, partially because of limited sample size, further research should aim for validation of these findings in independent larger prospective cohorts.

## 5. Conclusions

This study highlights the potential of inflammatory protein biomarkers for enhancing smoking-based prediction of lung cancer risk. We have identified inflammatory protein biomarkers that in combination with LC risk models enabled improved prediction of LC incidence. Models incorporating the inflammatory protein biomarkers along with established LC risk models may have important clinical implications for screening and preventive strategies. Further research should aim for further optimization of risk stratification algorithms and their validation in independent cohorts.

## Figures and Tables

**Table 1:** Characteristics of the ever smoking participants.

Characteristics	Training Set			Validation Set			Overall		
	Incident LC Cases *N* (%)	Random Participants Free of LC *N* (%)	*p* Value	Incident LC Cases *N* (%)	Random Participants Free of LC *N* (%)	*p* Value	Incident LC Cases *N* (%)	Random Participants Free of LC *N* (%)	*p* Value
	107	190		65	95		172	285	
Age (years)									
50–59	35 (33)	93 (49)	<0.01	18 (28)	46 (48)	<0.01	53 (31)	139 (49)	<0.01
60–69	56 (52)	78 (41)		37 (57)	36 (38)		93 (54)	114 (40)	
70–75	16 (15)	19 (10)		10 (15)	13 (14)		26 (15)	32 (11)	
Mean (SD)	62.2 (6.3)	60.2 (6.6)		63.1 (6.7)	60.7 (7.1)		62.5 (6.5)	60.4 (6.8)	
Median	62.0	60.0		63.0	60.0		62.0	60.0	
Gender									
Female	30 (28)	76 (40)	<0.05	20 (31)	31 (33)	0.86	50 (29)	107 (38)	0.06
Male	77 (72)	114 (60)		45 (69)	64 (67)		122 (71)	178 (62)	
Smoking status									
Former smoker	44 (41)	131 (69)	<0.01	21(32)	52 (55)	<0.01	65 (38)	183 (64)	<0.01
Current smoker	63 (59)	59 (31)		44 (68)	43 (45)		107 (62)	102 (36)	

Abbreviations: LC—lung cancer; *N*—number; SD—standard deviation.

**Table 2:** Performances for predicting LC incidence during 17 years of follow-up in discovery and validation sets among ever smoking participants of the ESTHER-study.

	Training Set N LC Cases—107 N LC-Free Participants—190		Validation Set N LC Cases—65 N LC-Free Participants—95	Proteins Included
	AUC*	AUC (95% CI)	AUC (95% CI)	
INS	0.770	0.771 (0.713–0.828)	0.742 (0.667–0.818)	CASP8, CCL11, CDCP1, CD8A, CD244, CXCL10, FGF19, MCP4, SCF
INS-pack-years	0.796	0.811 (0.760–0.863)	0.782 (0.711–0.854)	CASP8, CCL11, CCL25, CDCP1, CD8A, CD244, CXCL10, CXCL9, FGF19, MMP1

Abbreviations: AUC—area under the receiver operating curve; AUC*—0.632+ bootstrap adjusted estimate of area under the ROC curve; CASP8—caspase-8; CCL11—eotaxin; CCL25—C-C motif chemokine 25; CDCP1—CUB domain-containing protein 1; CD244—natural killer cell receptor 2B4; CD8A—T-cell surface glycoprotein CD8 alpha chain; CXCL10—C-X-C motif chemokine 10; CXCL9—C-X-C motif chemokine 9; FGF19—fibroblast growth factor 19; INS—inflammation protein biomarker score; INS-pack-years—combined inflammation protein biomarker and pack-years score; LC—lung cancer; MCP4—monocyte chemotactic protein 4; MMP1—matrix metalloproteinase-1; N—number; SCF—stem cell factor; 95% CI—95% confidence interval.

**Table 3:** Performances of different risk scores or models for predicting LC incidence during 17 years of follow-up in a case cohort design among ever smoking participants of the ESTHER-study.

Model	Training Set N LC Cases—107 N LC-Free Participants—190				Validation Set N LC Cases—65 N LC-Free Participants—95				Proteins Included
	AUC_LCmodel_ (95% CI)	AUC* AUC_LCmodel+INf_ (95% CI)	Improvement	*p* Val ^§^	AUC_LCmodel_ (95% CI)	AUC_LCmodel+INf_ (95% CI)	Improvement	*p* Val ^§^	
Bach [14]	0.765 (0.711–0.820)	0.807 * 0.811 (0.759–0.862)	0.042	0.24	0.752 (0.676–0.828)	0.770 (0.697–0.844)	0.018	0.73	CASP8, CDCP1, CD8A, CD244, CXCL10, FGF19, IL8
Spitz [15]	0.678 (0.614–0.743)	0.720 * 0.726 (0.666–0.786)	0.042	0.29	0.673 (0.589–0.756)	0.702 (0.622–0.782)	0.029	0.67	CDCP1, CXCL10, IL12B, IL8, SCF
LLP [16]	0.692 (0.629–0.756)	0.789 * 0.795 (0.740–0.849)	0.097	<0.05	0.703 (0.618–0.787)	0.756 (0.682–0.829)	0.053	<0.05	CASP8, CCL11, CDCP1, CD8A, CD244, CXCL10, FGF19, IL8, SCF
Hoggart [17]	0.738 (0.679–0.798)	0.791 * 0.800 (0.746–0.853)	0.053	0.13	0.700 (0.617–0.783)	0.745 (0.668–0.821)	0.045	0.44	CASP8, CCL11, CDCP1, CD8A, CD244, CXCL10, FGF19, IL8
PLCO_M2012_ [18]	0.669 (0.609–0.730)	0.790 * 0.791 (0.736–0.845)	0.121	<0.05	0.679 (0.594–0.763)	0.749 (0.672–0.825)	0.070	<0.05	CASP8, CCL11, CDCP1, CD8A, CD244, CXCL10, FGF19, IL8
LLPi [19]	0.736 (0.679–0.793)	0.746 * 0.747 (0.690–0.804)	0.010	0.79	0.734 (0.655–0.813)	0.743 (0.664–0.821)	0.009	0.89	CDCP1, CD244, IL12B, IL8
Pittsburgh Predictor [20]	0.767 (0.713–0.821)	0.800 * 0.801 (0.748–0.853)	0.033	0.38	0.784 (0.712–0.857)	0.794 (0.724–0.864)	0.010	0.86	CASP8, CDCP1, CD8A, CD244, CXCL10, IL8
LCRAT [21]	0.775 (0.722–0.829)	0.804 * 0.807 (0.756–0.859)	0.029	0.40	0.763 (0.687–0.841)	0.773 (0.700–0.845)	0.010	0.87	CASP8, CDCP1, CD8A, CD244, CXCL10, FGF19, IL8
LCDRAT [21]	0.770 (0.716–0.825)	0.781 * 0.785 (0.730–0.839)	0.011	0.71	0.766 (0.690–0.842)	0.775 (0.702–0.849)	0.009	0.86	CDCP1, CD244, CXCL10, IL12B, IL8

Abbreviations: AUC—area under the receiver operating curve; AUC*—0.632+ bootstrap adjusted estimate of area under the ROC curve; CASP8—caspase-8; CCL11—eotaxin; CDCP1—CUB domain-containing protein 1; CD244—natural killer cell receptor 2B4; CD8A—T-cell surface glycoprotein CD8 alpha chain; CXCL10—C-X-C motif chemokine 10; FGF19—fibroblast growth factor 19; INf—inflammatory protein biomarkers; IL8—interleukin 8; IL12B—interleukin 12 receptor subunit beta; LC—lung cancer; LCDRAT—Lung Cancer Death Risk Assessment Tool; LCRAT—Lung Cancer Risk Assessment Tool; LLP—Liverpool Lung Project Risk Model; LLPi—Liverpool Lung Project Incidence Risk Model; PLCO_M2012_—Prostate, Lung, Colorectal, and Ovarian Cancer Screening Trial Model 2012; *p* val—*p* value; SCF—stem cell factor; 95% CI—95% confidence interval. Note: *—denotes the 0.632+ bootstrap adjusted estimates of AUC; ^§^—denotes the *p* value presented from the DeLong test for assessing the differences in area under the receiver operating curves for the LC risk model only and the combined LC risk model + INf.

## Data Availability

The data that support the findings of this study are available from the corresponding author upon reasonable request.

## Supplementary Materials

The following supporting information can be downloaded at: https://www.mdpi.com/article/10.3390/cancers14092146/s1, Table S1: List of protein biomarkers in the Inflammation Panel; Table S2: Covariates included in the established Lung Cancer models and information based on questionnaire data; Table S3: Individual predictive performance of each of the 59 inflammatory protein biomarkers for predicting LC incidence; Table S4: Performances in form of AUC (95% CI), for predicting LC incidence by histological subtypes during 17 years of follow-up in discovery and validation sets among ever smoking participants of the ESTHER-study; Table S5: Proteins from signatures with their functions.

## Abbreviations

AUC	area under the ROC curve
AUC*	0.632+ bootstrap adjusted area under the ROC curve
CASP8	caspase-8
CCL11	eotaxin
CCL25	C-C motif chemokine 25
CDCP1	CUB domain-containing protein 1
CD244	natural killer cell receptor 2B4
CD8A	T-cell surface glycoprotein CD8 alpha chain
CXCL10	C-X-C motif chemokine 10
CXCL9	C-X-C motif chemokine 9
CRP	C-reactive protein
CV	coefficient of variance
ESTHER	Epidemiologische Studie zu Chancen der Verhütung, Früherkennung und optimierten Therapie chronischer Erkrankungen in der älteren Bevölkerung
FGF19	fibroblast growth factor 19
IL12B	interleukin 12 subunit beta
IL6	interleukin 6
IL8	interleukin 8
INS	inflammation protein biomarker score
INS-pack-years	combined inflammation protein biomarker and pack-years score
LC	lung cancer
LCDRAT	Lung Cancer Death Risk Assessment Tool
LCRAT	Lung Cancer Risk Assessment Tool
LLP	Liverpool Lung Project Risk Model
LLPi	Liverpool Lung Project Incidence Risk Model
LDCT	low-dose computed tomography
MMP1	matrix metalloproteinase-1
MCP4	monocyte chemotactic protein 4
N	number
PEA	proximity extension assay
PLCO_M2012_	Prostate, Lung, Colorectal, and Ovarian Cancer Screening Trial Model 2012
Q	quartile
QCC	quality control criteria
ROC	receiver operating characteristics
SCAD	smoothly clipped absolute deviation
SCF	stem cell factor
SD	standard deviation
95% CI	95% confidence intervals
