# Supplementary material for: Potential of Inflammatory Protein Signatures for Enhanced Selection of People for Lung Cancer Screening
Source: Cancers (Basel). 2022 Apr 26;14(9):2146. doi: 10.3390/cancers14092146 (PMC9103423; doi:10.3390/cancers14092146)
Supplement: Supplementary file 1 [file cancers-14-02146-s001.zip › cancers-1667977-supplementary.pdf]

Supplementary Table S1: List of protein biomarkers in the Inflammation Panel

Protein biomarker	Uniprot ID
Adenosine Deaminase (ADA)	P00813
Artemin (ARTN)	Q5T4W7
Axin-1 (AXIN1)	O15169
Beta-nerve growth factor (Beta-NGF)	P01138
C-C motif chemokine 19 (CCL19)	Q99731
C-C motif chemokine 20 (CCL20)	P78556
C-C motif chemokine 23 (CCL23)	P55773
C-C motif chemokine 25 (CCL25)	O15444
C-C motif chemokine 28 (CCL28)	Q9NRJ3
C-C motif chemokine 3 (CCL3)	P10147
C-C motif chemokine 4 (CCL4)	P13236
C-X-C motif chemokine 1 (CXCL1)	P09341
C-X-C motif chemokine 10 (CXCL10)	P02778
C-X-C motif chemokine 11 (CXCL11)	O14625
C-X-C motif chemokine 5 (CXCL5)	P42830
C-X-C motif chemokine 6 (CXCL6)	P80162
C-X-C motif chemokine 9 (CXCL9)	Q07325
Caspase-8 (CASP-8)	Q14790
CD40L receptor (CD40)	P25942
CUB domain-containing protein 1 (CDCP1)	Q9H5V8
Cystatin D (CST5)	P28325
Delta and Notch-like epidermal growth factor-related receptor (DNER)	Q8NFT8
Eotaxin (CCL11)	P51671
Eukaryotic translation initiation factor 4E-binding protein 1 (4E-BP1)	Q13541
Fibroblast growth factor 19 (FGF-19)	O95750
Fibroblast growth factor 21 (FGF-21)	Q9NSA1
Fibroblast growth factor 23 (FGF-23)	Q9GZV9
Fibroblast growth factor 5 (FGF-5)	Q8NF90
Fms-related tyrosine kinase 3 ligand (Flt3L)	P49771
Fractalkine (CX3CL1)	P78423
Glial cell line-derived neurotrophic factor (GDNF)	P39905
Hepatocyte growth factor (HGF)	P14210
Interferon gamma (IFN-gamma)	P01579
Interleukin-1 alpha (IL-1 alpha)	P01583
Interleukin-10 (IL-10)	P22301
Interleukin-10 receptor subunit alpha (IL-10RA)	Q13651
Interleukin-10 receptor subunit beta (IL-10RB)	Q08334
Interleukin-12 subunit beta (IL-12B)	P29460
Interleukin-13 (IL-13)	P35225
Interleukin-15 receptor subunit alpha (IL-15RA)	Q13261
Interleukin-17A (IL-17A)	Q16552
Interleukin-17C (IL-17C)	Q9P0M4
Interleukin-18 (IL-18)	Q14116
Interleukin-18 receptor 1 (IL-18R1)	Q13478
Interleukin-2 (IL-2)	P60568
Interleukin-2 receptor subunit beta (IL-2RB)	P14784
Interleukin-20 (IL-20)	Q9NYY1

Protein biomarker	Uniprot ID
Interleukin-20 receptor subunit alpha (IL-20RA)	Q9UHF4
Interleukin-22 receptor subunit alpha-1 (IL-22 RA1)	Q8N6P7
Interleukin-24 (IL-24)	Q13007
Interleukin-33 (IL-33)	O95760
Interleukin-4 (IL-4)	P05112
Interleukin-5 (IL-5)	P05113
Interleukin-6 (IL-6)	P05231
Interleukin-7 (IL-7)	P13232
Interleukin-8 (IL-8)	P10145
Latency-associated peptide transforming growth factor beta-1 (LAP TGF-beta-1)	P01137
Leukemia inhibitory factor (LIF)	P15018
Leukemia inhibitory factor receptor (LIF-R)	P42702
Macrophage colony-stimulating factor 1 (CSF-1)	P09603
Matrix metalloproteinase-1 (MMP-1)	P03956
Matrix metalloproteinase-10 (MMP-10)	P09238
Monocyte chemotactic protein 1 (MCP-1)	P13500
Monocyte chemotactic protein 2 (MCP-2)	P80075
Monocyte chemotactic protein 3 (MCP-3)	P80098
Monocyte chemotactic protein 4 (MCP-4)	Q99616
Natural killer cell receptor 2B4 (CD244)	Q9BZW8
Neurotrophin-3 (NT-3)	P20783
Neurturin (NRTN)	Q99748
Oncostatin-M (OSM)	P13725
Osteoprotegerin (OPG)	O00300
Programmed cell death 1 ligand 1 (PD-L1)	Q9NZQ7
Protein S100-A12 (EN-RAGE)	P80511
Signaling lymphocytic activation molecule (SLAMF1)	Q13291
SIR2-like protein 2 (SIRT2)	Q8IXJ6
STAM-binding protein (STAMPB)	O95630
Stem cell factor (SCF)	P21583
Sulfotransferase 1A1 (ST1A1)	P50225
T cell surface glycoprotein CD6 isoform (CD6)	Q8WWJ7
T-cell surface glycoprotein CD5 (CD5)	P06127
T-cell surface glycoprotein CD8 alpha chain (CD8A)	P01732
Thymic stromal lymphopoietin (TSLP)	Q969D9
TNF-beta (TNFB)	P01374
TNF-related activation-induced cytokine (TRANCE)	O14788
TNF-related apoptosis-inducing ligand (TRAIL)	P50591
Transforming growth factor alpha (TGF-alpha)	P01135
Tumor necrosis factor (Ligand) superfamily, member 12 (TWEAK)	O43508
Tumor necrosis factor (TNF)	P01375
Tumor necrosis factor ligand superfamily member 14 (TNFSF14)	O43557
Tumor necrosis factor receptor superfamily member 9 (TNFRSF9)	Q07011
Urokinase-type plasminogen activator (uPA)	P00749
Vascular endothelial growth factor A (VEGF-A)	P15692

In total, 33 biomarkers with > 1 % of the values below LOD (marked in grey) were excluded from all analyses.

Supplementary Table S2: Covariates included in the established Lung Cancer models and information based on questionnaire data

Covariate	Bach model	Spitz model	LLP model	Hoggart model	$PLCO_{M2012}$ model	Pittsburgh predictor	LLPi model	LCRAT model	LCDRAT model	Notes regarding application in ESTHER
**Age**	X	X	X	X	X	X	X	X		
**Gender**	X	X	X				X	X		
**Race/ethnicity**					X			X		Only Caucasian study participants
**Asian**					X					Not applicable in ESTHER, so coded 0
**Pacific islander**					X					Not applicable in ESTHER, so coded 0
**American Indian**					X					Not applicable in ESTHER, so coded 0
**BMI**					X			X		
**Education**					X			X		Education variable has six categories in risk models:<br>1=Less than grade 12; 2=high-school graduate;3=post high school but no college;4=some college;5=bachelor's degree;6= graduate school<br>In ESTHER, categories 4 to 6 are combined
**Smoking status**		X		X	X	X				
**Years/age quit**	X	X		X	X			X		
**Years smoked**	X		X	X	X	X	X	X		
**Cigs per day**	X			X	X	X		X		
**Pack-years**		X						X		
**Lung disease**		X			X		X	X		Lung disease denotes Emphysema or COPD<br>In Esther, information is available for Emphysema only
**Prior cancer**			X		X		X			
**Pneumonia**			X							
**Hayfever**		X								
**Asbestos exposure**	X	X	X							Not available in ESTHER, so coded 0
**Dust exposure**		X								
**Any FDR with cancer**		X								
**Any FDR with smoking related cancer**		X								
**Any FDR with LC**			X		X		X	X		
**Num. FDR with LC**								X		

**Early onset FDR with LC**			X				X			

Abbreviations: FDR- first degree relative; LC- lung cancer

Supplementary Table S3: Individual predictive performance of each of the 59 inflammatory protein biomarkers for predicting LC incidence

Protein biomarker	LC Mean	Cn Mean	AUC (95%CI)	p_value	p_value^{adj}
CDCP1	3.7	3.3	0.62 (0.57-0.67)	<0.001	0.001
CCL11	7.5	7.4	0.60 (0.54-0.65)	<0.001	<0.05
IL12B	4.3	4.5	0.60 (0.54-0.65)	<0.001	<0.05
TNFB	3.9	4.0	0.58 (0.52-0.63)	<0.05	0.08
CD244	6.3	6.4	0.58 (0.52-0.63)	<0.05	0.08
SCF	9.2	9.5	0.57 (0.52-0.63)	<0.05	0.08
CCL23	9.1	9.3	0.57 (0.51-0.62)	<0.05	0.12
TRANCE	4.2	4.3	0.57 (0.51-0.62)	<0.05	0.14
IL7	3.4	3.6	0.56 (0.51-0.61)	<0.05	0.20
CXCL10	9.0	9.2	0.56 (0.50-0.61)	<0.05	0.20
IL6	3.9	3.7	0.56 (0.50-0.61)	<0.05	0.24
CASP8	5.2	4.9	0.55 (0.50-0.61)	0.06	0.28
TWEAK	9.6	9.8	0.55 (0.50-0.61)	0.06	0.28
FGF21	5.1	5.0	0.55 (0.50-0.61)	0.07	0.29
CCL19	8.4	8.6	0.55 (0.50-0.60)	0.08	0.30
CX3CL1	5.9	6.1	0.55 (0.49-0.60)	0.09	0.33
HGF	9.5	9.4	0.54 (0.49-0.60)	0.11	0.39
CSF1	9.1	9.2	0.54 (0.49-0.60)	0.13	0.43
TRAIL	7.7	7.8	0.54 (0.48-0.60)	0.16	0.49
IL10RB	5.8	6.0	0.54 (0.48-0.59)	0.17	0.49
CD6	5.2	5.3	0.54 (0.48-0.59)	0.20	0.56
PD-L1	4.5	4.5	0.53 (0.48-0.59)	0.23	0.63
MCP1	11.7	11.6	0.53 (0.48-0.59)	0.28	0.68
CXCL11	7.8	8.0	0.53 (0.47-0.59)	0.28	0.68
MCP2	8.9	9.0	0.53 (0.47-0.58)	0.29	0.69
Flt3L	9.5	9.5	0.53 (0.47-0.58)	0.33	0.74
CD5	4.1	4.2	0.53 (0.47-0.58)	0.34	0.74
STAMPB	3.7	3.8	0.53 (0.47-0.58)	0.35	0.74
IL18R1	7.3	7.4	0.52 (0.47-0.58)	0.40	0.79
CCL4	7.3	7.2	0.52 (0.47-0.58)	0.43	0.79
CD8A	8.4	8.5	0.52 (0.47-0.58)	0.44	0.79
ADA	3.0	3.0	0.52 (0.47-0.58)	0.44	0.79
CD40	11.2	11.2	0.48 (0.42-0.54)	0.47	0.79
FGF19	7.6	7.7	0.52 (0.46-0.57)	0.48	0.79
MCP4	14.5	14.5	0.52 (0.46-0.57)	0.51	0.79
IL8	10.1	9.9	0.52 (0.46-0.57)	0.56	0.79
TNFRSF9	6.3	6.3	0.48 (0.43-0.54)	0.56	0.79
OSM	7.8	7.7	0.52 (0.46-0.57)	0.58	0.79
uPA	9.4	9.4	0.51 (0.46-0.57)	0.59	0.79
CXCL6	8.4	8.5	0.51 (0.46-0.57)	0.60	0.79
DNER	8.5	8.6	0.51 (0.46-0.57)	0.61	0.79
CCL20	5.0	4.9	0.51 (0.46-0.57)	0.63	0.79

Protein biomarker	LC Mean	Cn Mean	AUC (95%CI)	p_value	p_value^{adj}
CXCL1	10.2	10.2	0.51 (0.46-0.57)	0.63	0.79
Beta-NGF	1.1	1.2	0.49 (0.43-0.54)	0.63	0.79
LIFR	2.6	2.6	0.51 (0.46-0.57)	0.64	0.79
CXCL5	11.6	11.7	0.49 (0.43-0.54)	0.64	0.79
4E-BP1	6.1	6.2	0.51 (0.46-0.57)	0.64	0.79
EN-RAGE	5.9	5.9	0.51 (0.46-0.57)	0.65	0.79
CCL3	6.6	6.5	0.51 (0.46-0.57)	0.66	0.79
LAP-TGF-beta1	8.0	8.0	0.51 (0.46-0.57)	0.69	0.81
OPG	9.9	9.9	0.51 (0.45-0.56)	0.76	0.88
TGF-alpha	4.9	4.9	0.51 (0.45-0.56)	0.79	0.90
CXCL9	7.2	7.1	0.49 (0.44-0.55)	0.84	0.93
MMP10	6.2	6.1	0.50 (0.44-0.55)	0.86	0.93
VEGFA	10.5	10.5	0.50 (0.45-0.56)	0.86	0.93
TNFSF14	6.7	6.7	0.50 (0.44-0.55)	0.95	0.99
IL18	8.3	8.3	0.50 (0.44-0.55)	0.96	0.99
MMP1	14.4	14.5	0.50 (0.44-0.56)	0.97	0.99
CCL25	5.4	5.4	0.50 (0.44-0.55)	0.99	0.99

Abbreviations: AUC- area under the receiver operating curve; Cn- controls free of lung neoplasms; LC- lung cancer; p-value- apparent p-values without any adjustments; $p\text{-value}^{adj}$- p-value after adjustment for multiple testing by Benjamini Hochberg method; 95% CI- 95 % confidence interval.

Supplementary Table S4: Performances in form of AUC (95% CI), for predicting LC incidence by histological subtypes during 17 years of follow-up in discovery and validation sets among ever smoking participants of the ESTHER-study

	**Training set**<br>N LC cases - 107<br>N LC-free participants -190				**Validation set**<br>N LC cases - 65<br>N LC-free participants - 95			
	SCC<br>N- 33	AC<br>N- 44	UC<br>N- 18	SmCC<br>N- 12	SCC<br>N- 12	AC<br>N- 20	UC<br>N- 18	SmCC<br>N- 15
INS	0.76 (0.66-0.86)	0.76 (0.67-0.84)	0.80 (0.70-0.91)	0.79 (0.68-0.91)	0.66 (0.49-0.83)	0.79 (0.70-0.88)	0.71 (0.59-0.83)	0.79 (0.67-0.90)
INSPY	0.84 (0.77-0.92)	0.76 (0.68-0.85)	0.85 (0.76-0.93)	0.85 (0.77-0.93)	0.76 (0.59-0.94)	0.81 (0.72-0.90)	0.73 (0.62-0.84)	0.83 (0.73-0.93)

Abbreviations: AC- Adenocarcinoma; AUC- area under the receiver operating curve; INS- inflammation protein biomarker score; INS-pack-years- combined inflammation protein biomarker and pack-years score; N- number; SCC- Squamous cell carcinoma; SmCC- Small-cell carcinoma; UC- Unspecified carcinoma; 95% CI- 95 % confidence interval.

Supplementary Table S5: Proteins from signatures with their functions

GENE	UNIPROT ID	PROTEIN	MOLECULAR FUNCTION	BIOLOGICAL PROCESS
CASP8	Q14790	Caspase-8	hydrolase, protease, thiol protease	angiogenesis, host-virus interaction
CCL11	P51671	Eotaxin	cytokine	chemotaxis, inflammatory response
CCL25	O15444	C-C motif chemokine 25	cytokine	chemotaxis, inflammatory response
CDCP1	Q9H5V8	CUB domain-containing protein 1	cell adhesion and cell matrix association	inflammatory response
CD244	Q9BZW8	Natural killer cell receptor 2B4	receptor	adaptive immunity, immunity, innate immunity
CD8A	P01732	T-cell surface glycoprotein CD8 alpha chain	coreceptor activity	adaptive immunity, immunity
CXCL10	P02778	C-X-C motif chemokine 10	cytokine	chemotaxis, inflammatory response
CXCL9	Q07325	C-X-C motif chemokine 9	cytokine	inflammatory response
FGF19	O95750	fibroblast growth factor 19	growth factor	cell differentiation
IL8	P10145	Interleukin 8	cytokine	chemotaxis, inflammatory response
IL12B	P29460	Interleukin 12 receptor subunit beta	cytokine	inflammatory response
MCP4	Q99616	Monocyte chemotactic protein 4	cytokine	chemotaxis, inflammatory response
MMP1	P03956	Matrix metalloproteinase-1	hydrolase, metalloprotease	collagen degradation, host-virus interaction
SCF	P21583	Stem cell factor	growth factor	cell adhesion
